# Synthesis and Characterization of the New Manganese Hydroxide Chloride γ‐Mn(OH)Cl

**DOI:** 10.1002/ejic.201800928

**Published:** 2018-11-06

**Authors:** Viktoria Falkowski, Alexander Zeugner, Anna Isaeva, Michael Ruck, Hubert Huppertz

**Affiliations:** ^1^ Institut für Allgemeine Anorganische und Theoretische Chemie Leopold‐Franzens‐Universität Innsbruck Innrain 80‐82, A ‐6020 Innsbruck Austria; ^2^ Fakultät Chemie und Lebensmittelchemie Anorganische und Theoretische Chemie Technische Universität Dresden Bergstr. 66 01069 Dresden Germany

**Keywords:** High‐pressure synthesis, Hydroxide halide, Layered compounds, Magnetic properties, Manganese

## Abstract

A new modification of Mn(OH)Cl was obtained under high‐pressure/high‐temperature conditions in a Walker‐type multianvil device. The pale pink, hygroscopic compound crystallizes in the orthorhombic space group *Pnma* (no. 62) with *a* = 602.90(4), *b* = 350.98(2), *c* = 1077.69(7) pm, and *V* = 228 × 10^6^ pm^3^. The layered centrosymmetric structure consists of edge‐sharing Mn(OH)_3_Cl_3_ octahedra arranged in sheets parallel to the (001) plane. The comparatively long H**···**Cl distance of 275 pm suggests only weak hydrogen bonds between neighboring layers. Spin‐polarized scalar‐relativistic DFT+*U* calculations predict a non‐conducting magnetically ordered ground state with a band gap of at least 3.2 eV and an effective magnetic moment of 4.65 µ_B_/f. u. The experimentally determined magnetic response of Mn(OH)Cl is paramagnetic in the range of 10–300 K. The estimated moment of 5.6 µ_B_/f. u. indicates the high‐spin *d* ^5^ configuration of manganese(II). We find hints for a long‐range magnetic ordering below 10 K.

## Introduction

**Figure 1:**
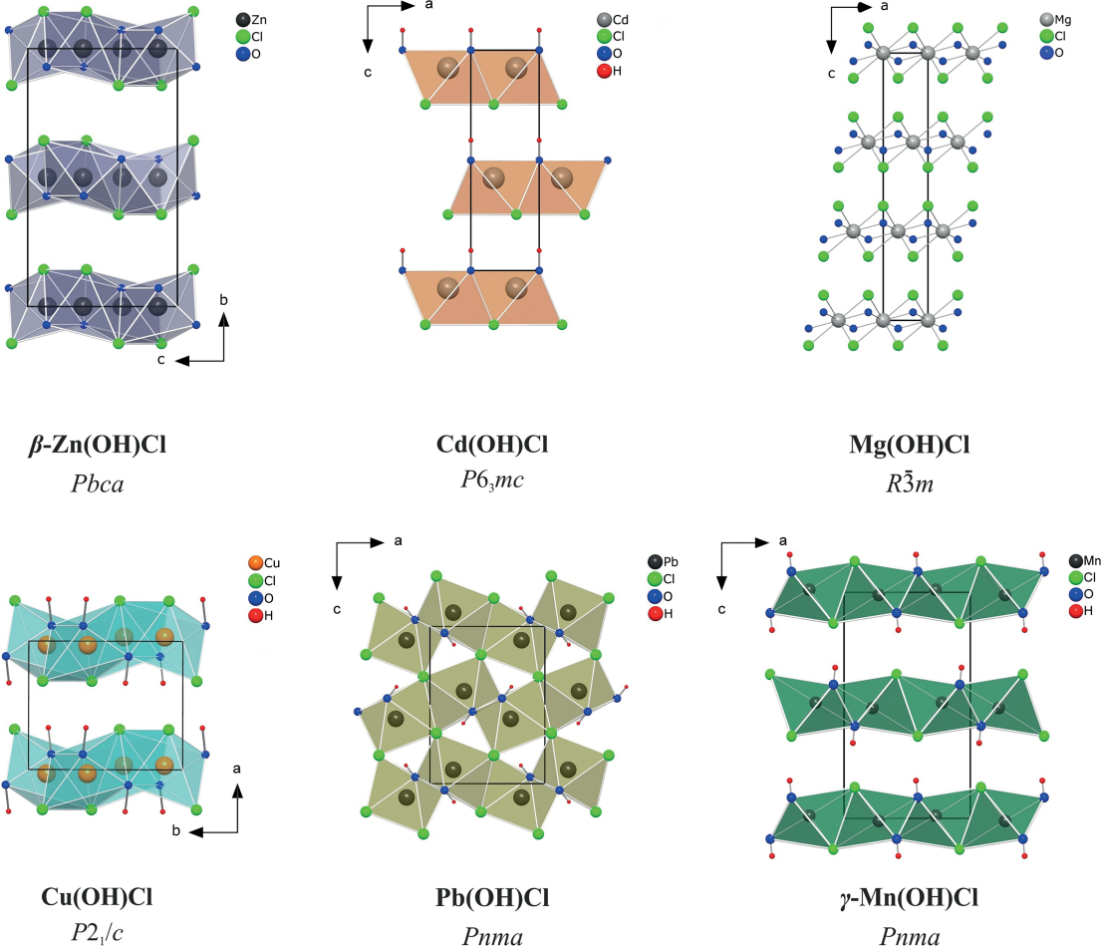
Crystal structure types of hydroxide chlorides of type M^II^(OH)Cl (black frames indicate the specific unit cell).

Numerous divalent metal hydroxide chlorides of composition M(OH)Cl with M = Mg, Ca, Ba, Sr, Pb, Mn, Fe, Co, Ni, Cu, Zn, Cd, and Sn are known so far. Not all of them could be structurally characterized by X‐ray single‐crystal or powder diffraction data yet.^1^, ^2^, ^3^, ^4^, ^5^, ^6^, ^7^, ^8^ These compounds crystallize with layered structures that can be derived from the Mg(OH)_2_ (brucite) or CdI_2_ structure type (C6) possessing a *hcp* stacking of the anions, or from the CdCl_2_ structure type (C19) with cubic close packing of the anions. In both cases, the cations occupy half of the octahedral voids. Figure 1 shows the crystal structure prototypes of the known hydroxide chlorides of type M^II^(OH)Cl (M = Zn, Cd, Mg, Cu, Pb) described below and the structure of the title compound. The most common structure types adapted by the structurally characterized divalent hydroxide chlorides are the types of β‐Zn(OH)Cl and Cd(OH)Cl.^2^ The β‐Zn(OH)Cl structure type crystallizes in an orthorhombic cell (space group *Pbca*) with layers of linked *mer*‐Zn(OH)_3_Cl_3_ octahedra. This structure type is adapted for example by Co(OH)*X* (*X* = Cl, Br).^9^ The hexagonal crystal structure of Cd(OH)Cl (space group *P*6_3_ *mc*), which is also found in the isotypic compounds Sr(OH)Cl^5^ and Ca(OH)Cl^2^ consists also of sheets of linked Cd(OH)_3_Cl_3_ octahedra. But in this case, chloride and hydroxide anions are separated on opposite faces of the layer. The remaining hydroxide chloride structures crystallize in the Cu(OH)Cl,^6^ Mg(OH)Cl,^7^ and Pb(OH)Cl^4^ structure types. The layered mineral belloite [Cu(OH)Cl] crystallizes in the monoclinic space group *P*2_1_/*c* with an equal distribution of chloride anions and hydroxide groups in each anion layer. The distorted octahedral coordination around the copper atom can be seen as a [4+2] coordination with two opposing chloride anions at larger distances to the cation compared to the single chloride anion in the equatorial position. In the trigonal crystal structure of Mg(OH)Cl (space group *R* 3 *m*), chloride anions and hydroxide groups are statistically distributed within the Mg(OH)_3_Cl_3_ octahedra. The crystal structures of Pb(OH)Cl and the isotypic compound Ba(OH)Cl^8^ represent exceptions among the hydroxide chlorides as they consist of three‐dimensional networks rather than layers separated by van der Waals gaps. Though some compounds of this class were structurally characterized, a combination of poor crystallinity, occurrence of polymorphism, stacking disorder, and a strong hygroscopicity challenges a detailed analysis of these phases.

Such a case is presented by the compound Mn(OH)Cl. Already synthesized and described by Ribi in 1948,^10^ compounds with these composition are still not entirely structurally elucidated. Cell parameters of the orthorhombic α‐phase were determined, but they disagree in the different references. In a work from 1959, Feitknecht et al. reported the cell parameters *a* = 607, *b* = 691, and *c* = 1149 pm.^1^ The values of the parameters *a* and *b* correspond well with the given values in the work published two years later by Feitknecht and Oswald.^2^ The *c* value given in the first reference (1146 pm) though differs by a factor of three from the one published in 1961 (3455 pm). For the β‐form, the cell parameters reported in the second work are very similar to those of α‐Mn(OH)Cl, except crystallizing in a hexagonal crystal system. For both phases, no specific details on their crystal structures are available. The colorless α‐form was obtained by reaction of manganese metal in a saturated MnCl_2_ solution under exclusion of oxygen followed by annealing the product at 383 to 423 K. Treatment of this compound at temperatures of 573 to 593 K led to the formation of the pale rose‐colored β‐Mn(OH)Cl. Both forms are described as hygroscopic and oxidation‐sensitive phases with a strong tendency for structures with stacking faults.

Since accurate structural data for these compounds are lacking, the aim of this work was the synthesis of manganese hydroxide chloride phases by means of high‐pressure/high‐temperature syntheses. Due to the fact that such conditions during the synthesis can improve the crystallinity and quality of the crystals, the possibility to obtain crystals suitable for a single‐crystal X‐ray structure determination was evaluated. In the course of these inquiries, a new modification of Mn(OH)Cl was discovered and is designated as γ‐Mn(OH)Cl in the following.

## Results and Discussion

### Synthesis

The compound γ‐Mn(OH)Cl was obtained at 573 K and 2.5 GPa. However, best results regarding crystallinity and phase purity were received by heating the sample to 873 K after compression. This temperature was reached within 5 min, maintained for 25 min, then decreased to 573 K within 30 min, held for 90 min, reduced to 373 K within 4 h, and subsequently quenched to room temperature.

A hygroscopic, pale‐rose colored phase could be isolated. The compound was stored in a glovebox, though larger crystals showed a reasonable air stability and did not require handling in an inert atmosphere. Single‐crystals appeared colorless, exhibiting a platy habit with a tendency for building stacks of twin layers.

### Crystal Structure

The manganese hydroxide halide γ‐Mn(OH)Cl crystallizes with four formula units (*Z* = 4) in the orthorhombic space group *Pnma* (no. 62) with the lattice parameters *a* = 602.90(4), *b* = 350.98(2), *c* = 1077.69(7) pm at 173(2) K (Table 1). Table 2 and Table 3 contain positional parameters, anisotropic displacement parameters, and selected interatomic distances. Bond angles are listed in Table S1.

**Table 1:** Crystal data and structure refinement of γ‐Mn(OH)Cl (standard deviations in parentheses)

Molar mass [g mol^–1^]	107.40
Crystal system	orthorhombic
Space group	*Pnma* (no. 62)
Powder data	
Powder diffractometer	Stoe Stadi P
Radiation	Mo‐*K_α_*_1_ (*λ* = 70.930 pm)
Temperature [K]	293
*a* [pm]	603.81(2)
*b* [pm]	351.49(2)
*c* [pm]	1079.31(4)
*V* [10^6^ pm^3^]	229.07
Single‐crystal data	
Single‐crystal diffractometer	Bruker D8 Quest
Radiation	Mo‐*K_α_* (*λ* = 71.073 pm)
Temperature [K]	173(2)
*a* [pm]	602.90(4)
*b* [pm]	350.98(2)
*c* [pm]	1077.69(7)
*V* [10^6^ pm^3^]	228.05
Formula units per cell	*Z *= 4
Calculated density [g cm^–3^]	3.13
Absorption coefficient [mm^–1^]	6.5
Crystal size [mm^3^]	0.020 × 0.070 × 0.070
2*θ* range [°]	7.6–69.9
Range in *hkl*	–9 ≤ *h* ≤ 9, –5 ≤ *k* ≤ 5, –17 ≤ *l* ≤ 17
Total no. of reflections	9593
Independent reflections	569 (*R*_*i*n*t*_ = 0.035)
Reflections with *I* ≥ 2σ(*I*)	534 (*R*_σ_ = 0.012)
Data/restraints/parameters	569/1/22
Goodness‐of‐fit on *F* ^2^	1.090
Final *R* indices [*I* ≥ 2σ(*I*)]	*R*1 = 0.017, *wR*2 = 0.038
*R* indices (all data)	*R*1 = 0.019, *wR*2 = 0.039
Largest diff. peak and hole [e 10^–6^ pm^3^]	0.56/–0.53

**Table 2:** Atomic coordinates, equivalent isotropic displacement parameters (*U*_eq_/pm^2^) and anisotropic displacement parameters (*U*_ij_/pm^2^) of γ‐Mn(OH)Cl (space group *Pnma*). *U*_eq_ is defined as one third of the trace of the orthogonalized *U*_ij_ tensor. All atoms occupy Wyckoff position 4*c; U*_23_ = *U*_12_ = 0 (standard deviations in parentheses)

Atom	*x*	*y*	*z*	*U*_eq_	*U*_11_	*U*_22_	*U*_33_	*U*_13_
Mn	0.77512(4)	3/4	0.48831(2)	92.5(7)	64(1)	66(2)	148(2)	–8.6(7)
Cl	0.41538(6)	3/4	0.36340(3)	99.6(8)	91(2)	97(2)	111(2)	–3(2)
O	0.9165(2)	1/4	0.4083(2)	87(2)	84(4)	88(4)	89(4)	0(3)
H	0.930(4)	1/4	0.3331(9)	120(50)	–	–	–	–

**Table 3:** Interatomic distances (/pm) in γ‐Mn(OH)Cl, calculated with the single‐crystal lattice parameters (standard deviations in parentheses)

Mn – O	213.30(6)	Mn – Mn^v^	323.97(3)
Mn – O^i^	213.30(6)	Mn – Mn^ii^	323.97(3)
Mn – O^ii^	216.7(2)	Mn – Mn^vi^	350.98(2)
Mn – Cl	255.27(4)	Mn – Mn^i^	350.98(2)
Mn – Cl^iii^	263.68(3)	Mn – Mn^iv^	376.14(4)
Mn – Cl^iv^	263.68(3)	Mn – Mn^iii^	376.14(4)
			
O – Mn	213.30(6)	Cl – Mn	255.27(4)
O – Mn^vi^	213.30(6)	Cl – Mn^iii^	263.68(3)
O – Mn^ii^	216.7(2)	Cl – Mn^iv^	263.68(3)
O – H	81.5(9)		

(i) *x*, 1 + *y, z*;

(ii) 2 – *x*, 0.5 + *y*, 1 – *z*;

(iii) 1 – *x*, 0.5 + *y*, 1 – *z*;

(iv) 1 – *x*, –0.5 + *y*, 1 – *z*;

(v) 2 – *x*, –0.5 + *y*, 1 – *z*;

(vi) *x*, –1 + *y, z*.

**Figure 2:**
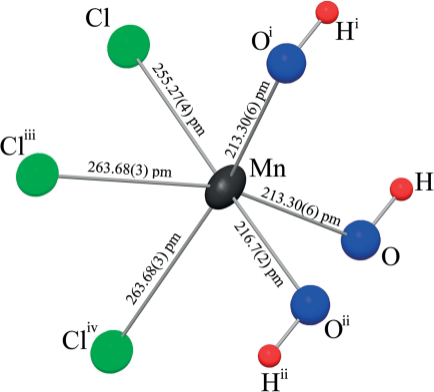
Distorted octahedral coordination around the manganese atom with facial arrangement of the ligands (thermal ellipsoids comprise 90 % of the probability density for non‐hydrogen atoms at 173 K).

**Figure 3:**
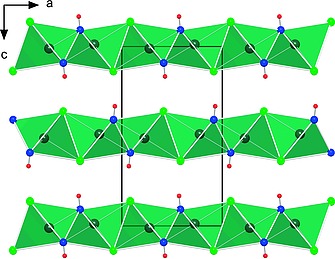
Layers of edge‐sharing Mn(OH)_3_Cl_3_ octahedra parallel to the *ab*‐plane viewed along the *b*‐axis (black frame indicates the unit cell).

The structure of the hydroxide chloride can be derived from the CdCl_2_ structure type with the metric relation to the trigonal structure *a/b* = 1.718, which is close to √3 = 1.732 for the orthohexagonal setting. The layers consist of edge‐sharing distorted octahedra, formed by three hydroxide groups and three chloride anions around a manganese(II) atom opposing each other (Figure 2 and Figure 3). The Mn–Cl distances of 264 pm (2×) and 255 pm (1×) are longer than in MnCl_2_ (254 pm^11^). The three oxygen atoms of the hydroxide groups are at 213 pm (2×) and 217 pm (1×) and thus shorter than the Mn–O distance of 220 pm in Mn(OH)_2_.^12^ This indicates that hydroxide is a stronger ligand to manganese(II) than chloride.

The bond angles at the central atom strongly deviate from 90° or 180° with Cl–Mn–Cl angles of 87° (2×) and 83°, O–Mn–O = 82° (2×) and 111°, and O–Mn–Cl*_trans_* = 165° (2×) and 179°.

**Figure 4:**
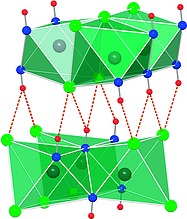
Bifurcated hydrogen bonds of the type O–H**···**Cl connecting two adjacent layers.

Adjacent layers are connected by equidistant bifurcated hydrogen bonds of the type O–H**···**Cl with an O–H–Cl angle of 140° (Figure 4). The restrained refinement of the O–H distance in γ‐Mn(OH)Cl results in the value of 82 pm with an interlayer O**···**Cl distance of 341 pm and an H**···**Cl distance of 275 pm. The latter value is close to the limit of 280 pm for hydrogen bonds of the type H**···**Cl proposed by Peter et al.^13^ indicating that there are only weak attractive interactions between the layers.

**Figure 5:**
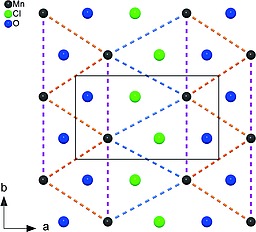
Single layer in γ‐Mn(OH)Cl viewed along the *c*‐axis (oxygen atoms represent hydroxide groups). Colored dashed bonds indicate different Mn–Mn distances [orange: 323.97(3) pm; pink: 350.98(2) pm; blue: 376.14(4) pm; the unit cell is outlined].

Examining the individual layers in the hydroxide chloride, three different intralayer Mn–Mn distances (324, 351 and 376 pm) are apparent, resulting from the connection of the asymmetric octahedra (Figure 5). The intermediate distance (pink) can be found in the direction of the Cl,O‐shared edges, hence aligned parallel to [010]. The longest (blue) and the shortest bonds (orange) are in the direction of the shared edges either with Cl or O on both corners, alternating in the pseudo‐trigonal directions.

Since we found isotypic representatives neither in this class of hydroxide halides nor among other compound classes, we consider γ‐Mn(OH)Cl as a new structure type.

### ATR‐IR Spectroscopy

**Figure 6:**
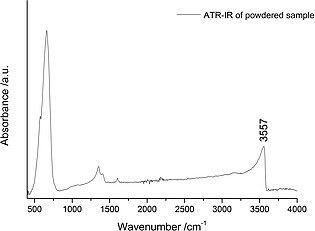
FTIR‐ATR spectrum of powdered γ‐Mn(OH)Cl sample.

Figure 6 shows the experimental IR spectrum of a powdered sample of γ‐Mn(OH)Cl in the range from 400 to 4000 cm^–1^. The band at 3557 cm^–1^ can be assigned to the ν(O–H) stretching vibration and confirms the existence of the hydroxide group in this structure. The slightly blue‐shifted band compared to the average value for a free hydroxide group [ν(O–H) = 3620 cm^–1[14]^] confirms the existence of weak hydrogen bonds of the type O–H**···**Cl. The broad band from 3000 to 3300 cm^–1^ is due to adsorbed water and the weak band at 1600 cm^–1^ can be attributed to the δ‐H_2_O bending vibration of the O–H groups in the adsorbed water molecules. Compared to the works of Ananth et al.^15^ and Kohler et al.,^16^ the peaks at 1353 and 1400 cm^–1^ are presumably due to the coordination of Mn by the O–H groups and the weak band from 1000 to 1200 cm^–1^ is considered to be a result of O–H bending modes of the hydroxide group. The strong band from 500 to 750 cm^–1^ can be assigned to Mn–O stretching modes.

### Electronic Structure

A self‐consistent calculation without spin‐polarization (so called non‐magnetic, NM) yields a gapped electronic spectrum with *E*_g_ = 0.44 eV. We find that any magnetically ordered scenario is energetically far more stable than the NM state (Δ*E* = *E*_FM_ – *E*_NM_ = 2.5 eV/f. u.). Simultaneously, the estimated energy differences between the various magnetic ordering patterns (see below) have a magnitude of several meV, so that the most preferred magnetic arrangement in γ‐Mn(OH)Cl cannot be unambiguously inferred. In the following, we study various scenarios.

**Figure 7:**
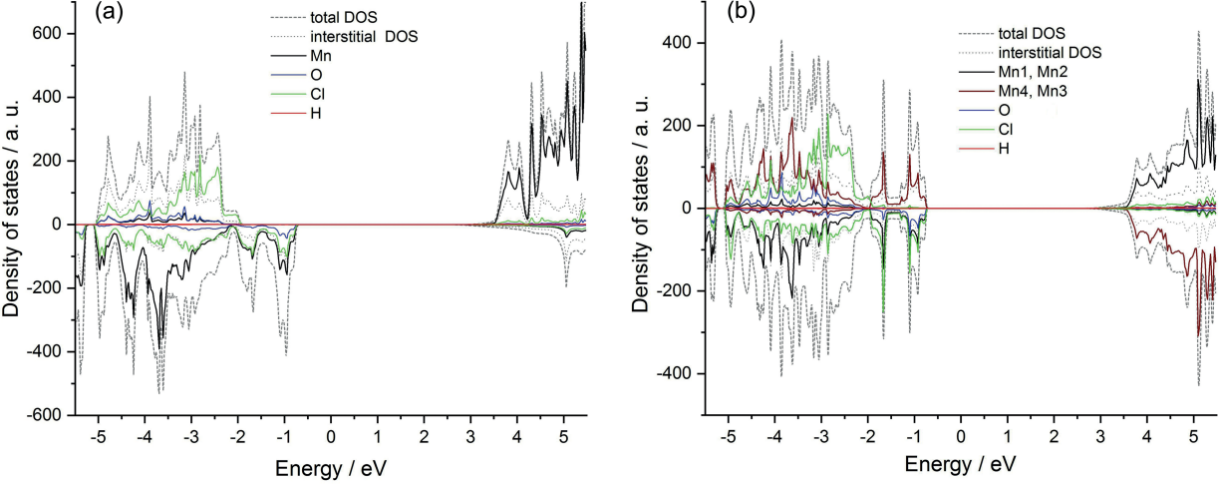
Total and partial density of states for majority (positive values) and minority (negative values) spin states in (a) the ferromagnetically ordered γ‐Mn(OH)Cl and (b) the antiferromagnetically (AFM3 model) ordered γ‐Mn(OH)Cl. Atomic coordinates: Mn1 0.7751 0.75 0.4883; Mn2 0.2249 0.25 0.5117; Mn3 0.7249 0.25 0.9883; Mn4 0.2751 0.75 0.0117.

The ferromagnetic state of γ‐Mn(OH)Cl has an increased total band gap of ca. 3.21 eV and the net magnetic moment 4.66 _B_/f. u. The gap size accords well with the observed almost transparent appearance of the crystals with a slight pinkish hue. A sizeable gap of 4.62 eV appears at the Fermi energy for the majority spins (Figure 7a). For the minority spin states, a gap of almost 3 eV is found. The populated Mn‐*d* states are strongly hybridized with the O‐*p* and Cl‐*p* states up to –2 eV below the Fermi edge, which is in full consistency with covalent bonding. Deviation of the calculated total magnetic moment from the idealized “spin‐only” value may be caused by partial delocalization of the manganese(II) spin density onto polarizable Cl and O states due to covalency effects. The empty Mn‐*d* states with an opposite spin are separated by the band gap. Prevailing contribution of the localized Mn‐*d* states is found between –5 eV and –2 eV.

The crystal structure provides many possibilities to realize antiferromagnetic intralayer couplings between the adjacent Mn atoms (Figure 5). Magnetic frustration on a triangular lattice is, however, unlikely due to differing interatomic distances. On top of that, the interlayer magnetic coupling could be realized in various ways (cf. Figure 3). We have considered only three scenarios involving AFM couplings that did not require increased unit‐cell volume. In other words, only the symmetry elements connecting the Mn atoms within a unit cell were broken. The most straightforward way is to keep ferromagnetic intralayer coupling and to introduce the antiferromagnetic interlayer coupling (AFM1). This type of magnetic ordering is by 3 meV/unit cell less stable than the FM state. Three distinct intralayer Mn–Mn interatomic distances enable several variations of nearest‐neighbour AFM magnetic exchange (Figure 5). We do not consider an arrangement, in which the interactions marked in orange or pink color are ferromagnetic, and those in blue are antiferromagnetic, since this case requires a superstructure. If the Mn spins point in opposite directions across the blue line, those connected by orange‐colored lines must be antiferromagnetically aligned and those along the pink lines have parallel orientation. In the AFM2 model, such sheets couple antiferromagnetically along the stacking direction. This type of magnetic ordering is by 2.5 meV/unit cell less stable than the FM state. The AFM3 model includes a ferromagnetic interlayer coupling (Figure 7b).

All listed AFM schemes result in an insulating state with a 3.4 eV gap and a magnetic moment of 4.65 µ_B_ per manganese(II) atom. Energy differences between them are vanishingly small, yet the AFM3 scenario is the most energetically preferable out of the considered three options. The total energy calculation shows that the AFM3 arrangement is energetically even more stable than the ferromagnetic one, but the energy difference Δ*E* = *E*_FM_ – *E*_AFM3_ = 4 meV/unit cell is very small.

In the AFM3 configuration (Figure 7b), strong intermixing of the Mn‐*d*, O‐*p*, and Cl‐*p* states in the vicinity of the Fermi level over an extended energy range of several eV reflects strong covalent bonding.

Due to rapidly growing complexity of further possible long‐range magnetic ordering schemes including canted spin configurations and superstructures, we did not engage into further simulations. Additionally, GGA treatment of the exchange‐correlation functional was implemented. The results demonstrate the slightly higher energy stability of the AFM (Δ*E* = *E*_FM_ – *E*_AFM_ = 7 meV/unit cell) and AFM3 (Δ*E* = *E*_FM_ – *E*_AFM3_ = 1 meV/unit cell) arrangements over the ferromagnetic one as well as the significant preference of the magnetic ordering over the “non‐magnetic” state (Δ*E* = *E*_FM_ – *E*_NM_ = 3.8 eV/f. u.). The AFM2 model is only by 0.5 meV/unit cell less favorable than the FM model. The calculated magnetic moment is 4.67 µ_B_/f. u. for all considered ordering types. Furthermore, careful lattice geometry optimization would help to analyze the energy differences between the magnetically ordered states on a higher level. These extensions lie out of scope of the present preliminary study.

Nevertheless, it can be concluded that a magnetically ordered insulating ground state is much more energetically stable in γ‐Mn(OH)Cl than a non‐interacting paramagnetic one. However, the lowest‐energy state was likely not determined in this work and may accommodate a complex noncollinear spin structure. Close energies of various antiferromagnetic and ferromagnetic out‐of‐plane arrangements may hint at competing magnetic ground states or a temperature‐dependent change of magnetic ordering.

### Magnetization Measurements

**Figure 8:**
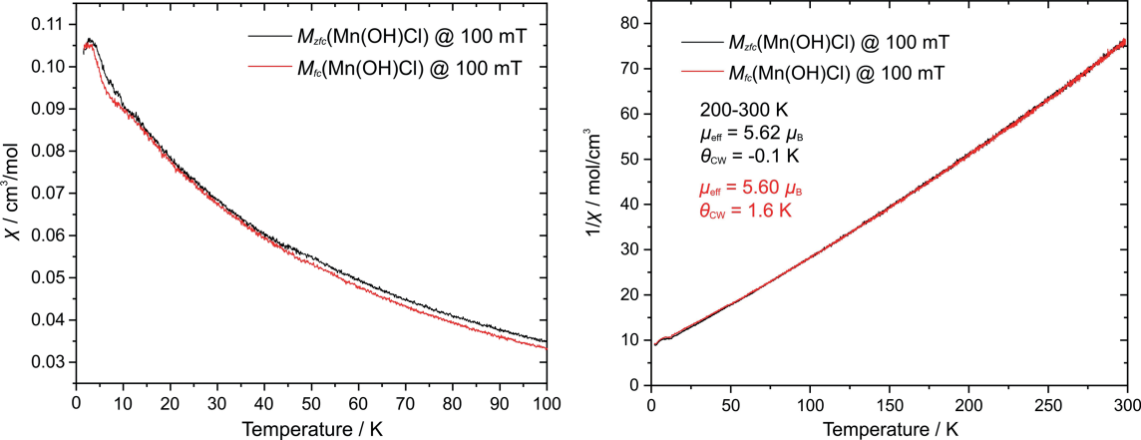
Left: Temperature‐dependent magnetic susceptibility measurements of γ‐Mn(OH)Cl in the *zfc* and *fc* regimes. Right: The corresponding temperature‐dependent inverse magnetic susceptibilities with the results of the Curie–Weiss fits of the *zfc* (black) and *fc* (red) curves in the 200–300 K range.

**Figure 9:**
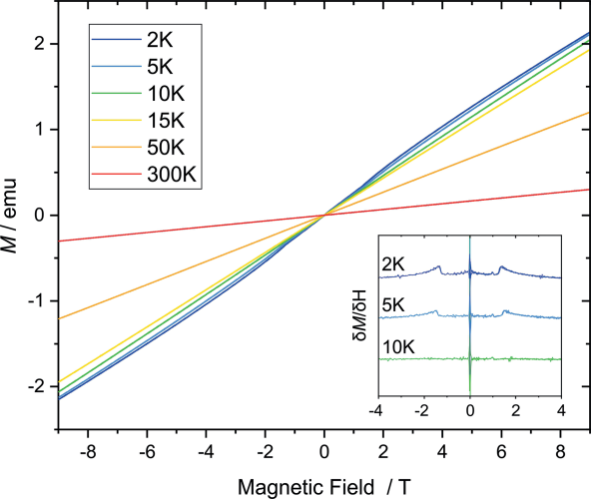
Field‐dependent magnetization measurements of γ‐Mn(OH)Cl at various temperatures. The derivative is shown in the inset in order to highlight the possible spin‐flop transition.

Temperature‐ and field‐dependent magnetization curves were acquired from a powder sample of γ‐Mn(OH)Cl (Figure 8, Figure 9). Single‐crystals were picked up individually, but the chemical purity of the sample might have been compromised due to high moisture‐sensitivity. Paramagnetic behavior was found in a broad temperature range from 300 to 10 K. The effective magnetic moment of ca. 5.6 µ_B_ was estimated from a linear Curie–Weiss fit of the zero‐field‐cooled warming (*zfc*) and field‐cooled warming (*fc*) curves in the temperature range 200–300 K. The estimated paramagnetic Curie temperature *θ*_CW_ is close to zero and is likely indicative of competing ferro‐ and antiferromagnetic exchange couplings in the system. A slight mismatch between the *zfc* and fc measurements could point at some short‐range magnetic correlations in the paramagnetic state. Moreover, a broadened feature centered at ca. 4 K is observed on the magnetization curves for *zfc* and *fc* sweeps. Upon cooling down to 2 K, the susceptibility starts to decrease, but further data acquisition was unavailable due to the device limitations. Furthermore, the *M*(*H*) curves of γ‐Mn(OH)Cl detect what could be a spin‐flop transition at 2 T below 10 K, which is consistent with the temperature‐dependent susceptibility. This scope of data may indicate a transition into a magnetically ordered state in γ‐Mn(OH)Cl. But the chemical purity of the sample could not be rigorously controlled, and the presence of magnetic impurities could not be refuted.

The elucidated magnetic characteristics of γ‐Mn(OH)Cl can be discussed in the context of selected compounds with manganese(II) atoms octahedrally coordinated by Cl and/or O. In general, the “spin‐only“ value is expected for the magnetic moment in the *d* ^5^ configuration. MnO orders antiferromagnetically at about 118 K, and its experimental magnetic moment of 4.58–4.79 µ_B_ is well reproduced by theory.^17^ The notable deviation from the “spin‐only“ moment may be attributed to covalency effects. As opposed to that, paramagnetic MnCl_2_ with the layered CdCl_2_‐type structure appears to be much closer to the idealized “spin‐only“ case with the experimental moment of 5.73 µ_B_/f. u.^18^ The observed behavior cannot be readily explained in the view of a quite moderate difference in the electronegativity of oxygen and chlorine. Moreover, anhydrous MnCl_2_ has a slightly negative *θ*_CW_ = –3.3 K and undergoes two magnetic phase transitions below 2 K. The magnetic structures are very complex, probably incommensurately helimagnetic, and have not been completely elucidated by now.^19^ Our test calculation of a simplified AFM model for MnCl_2_ yields the net moment of 4.75 µ_B_/f. u. The significant discrepancy between the experimental and calculated magnetic moments in γ‐Mn(OH)Cl may, in a similar way, be a result of an oversimplified model of the magnetic ordering. Sheets of edge‐sharing [MnCl_6_] octahedra in MnCl_2_ bear certain similarity to those in γ‐Mn(OH)Cl, and, therefore, could account for comparable exchange pathways.

Strong propensity to AFM ordering is also observed in the chain‐like compounds with mixed Cl/O octahedral coordination of the divalent Mn. In MnCl_2_(urea)_2_, the manganese(II) atom is octahedrally coordinated by four chloride anions in the equatorial plane and two apical oxygen atoms from urea ligands. The edge‐sharing octahedra form chains with AFM intrachain and FM interchain couplings. Like in γ‐Mn(OH)Cl, a broad maximum at 9 K on the *χ*(*T*) curve appears in MnCl_2_(urea)_2_ and can be attributed to a short‐range magnetic ordering between divalent Mn cations.^20^ High‐field magnetization data in MnCl_2_(urea)_2_ yield a saturated magnetic moment of 5.3 ± 0.2 µ_B_/f. u., whereas the zero‐field magnetic structure features the total magnetic moment of 4.06(6) µ_B_ per manganese(II), probably due to diffusion of the spin density onto the chloride ligands. Thus, the influence of covalent bonding can account for deviations of the magnetic moment from the “spin‐only“ value.

The same AFM‐chain model is applicable to MnCl_2_ **·**2H_2_O that undergoes a transition at *T*_N_ = 6.9 K and exhibits the same distorted [4+2] octahedral coordination of manganese(II) by Cl and O atoms. It is emphasized that hydrogen‐bonded pathways between the chains are important for large interchain magnetic exchange.^21^ In a similar fashion, extensive amount of hydrogen bonding may be relevant for long‐range magnetic ordering in γ‐Mn(OH)Cl. The magnetic moments of hydrated MnCl_2_ **·** *x*H_2_O are again very close to the expected “spin‐only“ value of 5.92 µ_B_ for the divalent Mn.^22^

## Conclusions

A new modification of the manganese hydroxide chloride Mn(OH)Cl was successfully synthesized via high‐pressure/high‐temperature synthesis in a multianvil device. As the α‐ and β‐phase are still not fully structurally characterized, it is the first compound with this composition to be structurally elucidated. The new compound assigned as γ‐Mn(OH)Cl consists of linked Mn(OH)_3_Cl_3_ octahedra forming sheets with weak interlayer hydrogen bonding, which is verified by IR spectroscopy. Its unique structure constitutes a new representative of the material class of layer‐type hydroxide chlorides, extending their chemical and structural variety. A preliminary DFT+*U* study predicts a magnetically ordered insulating ground state for γ‐Mn(OH)Cl with a sizeable band gap above 3.2 eV and a local magnetic moment of 4.65 µ_B_/f. u. Competing FM and AFM magnetic interactions may underlie the very small energy differences between the various modelled spin arrangements and the very small value of the experimental Curie paramagnetic temperature close to zero. The effective paramagnetic moment of ca. 5.6 µ_B_/f. u. is close to the “spin‐only” value expected for the high‐spin manganese(II) configuration. There are experimental indications of a transition likely to a magnetically ordered state below 10 K that are in line with the observations for MnCl_2_, MnCl_2_ **·** *x*H_2_O, and MnCl_2_(urea)_2_ with complex magnetic structures and similar octahedral coordination of the divalent manganese atom.

## Experimental Section

**Synthesis:** Single‐crystals of the manganese hydroxide chloride γ‐Mn(OH)Cl were obtained under high‐pressure/high‐temperature conditions from a 1:1 reaction mixture of Mn (99+%, Sigma Aldrich, Steinheim, Germany) and MnCl_2_ **·**4H_2_O (*purum*, p. a., Fluka, Buchs, Switzerland). Therefore, the finely ground reaction mixture was filled into a molybdenum capsule (0.025 mm foil, 99.95 %, Alfa Aesar, Karlsruhe, Germany) and transferred into a crucible made from hexagonal boron nitride (HeBoSint® P100, Henze BNP GmbH, Kempten, Germany). This crucible was built into an 18/11 assembly and afterwards compressed by eight tungsten carbide cubes (HA‐7 %Co, Hawedia, Marklkofen, Germany). Additional information on the construction of the assembly can be found in the corresponding reference.^23^ The pressure was applied by a hydraulic press (mavo press LPR 1000–400/50, Max Voggenreiter GmbH, Mainleus, Germany) and a modified Walker‐type module (also Max Voggenreiter GmbH).

**X‐ray Structure Determination:** For characterization by X‐ray powder diffraction, the sample was placed in a 0.7 mm glass capillary (Hilgenberg, Malsfeld, Germany) and data was collected with a Stoe Stadi P powder diffractometer in Debye–Scherrer geometry and Mo‐*K*_*α*1_ (*λ* = 70.930 pm) radiation applying a focusing Ge(111) primary beam monochromator and a Mythen2 1K detector (Dectris, Baden, Switzerland). The lattice parameters were refined using the software TOPAS^24^ to compare to the values derived from the single‐crystal structure determination.

For a single‐crystal structure determination, suitable crystals of γ‐Mn(OH)Cl covered in protective perfluoropolyalkylether oil (viscosity 1800 cSt, ABCR chemicals, Karlsruhe, Germany) were isolated under a polarization microscope. Intensity data was collected at 173 K with a Bruker D8 Quest diffractometer equipped with a Photon 100 detector and an Incoatec Microfocus source generator (multi layered optics monochromatized) using Mo‐*K_α_* radiation (*λ* = 71.073 pm). Multi‐scan absorption corrections based on equivalent and redundant intensities were applied with the program Bruker Sadabs‐2014/5.^25^, ^26^ The hydrogen atom was refined with an isotropic displacement parameter, restraining the O–H distance to a value of 83 ± 2 pm. All non‐hydrogen atoms were refined anisotropically, resulting in *R*1 and *wR*2 values of 0.019 and 0.039 (all data). Relevant experimental details of the data collection and evaluations are summarized in Table 1.

CCDC 1856185 (for **y‐Mn(OH)Cl**) contains the supplementary crystallographic data for this paper. These data can be obtained free of charge from The Cambridge Crystallographic Data Centre.

**IR Spectroscopy:** To confirm the hydroxide group and to receive information about hydrogen bonding, a FTIR‐ATR (Attenuated Total Reflection) spectrum of the compound was acquired. Therefore, a powdered sample of γ‐Mn(OH)Cl was measured with a Bruker ALPHA Platinum‐ATR spectrometer (Bruker, Billerica, USA) with a diamond ATR‐crystal (2 × 2 mm), equipped with a DTGS detector in the spectral range of 400–4000 cm^–1^ (spectral resolution 4 cm^–1^). 24 scans of the sample were acquired and a correction for atmospheric influences using the opus 7.2^27^ software was performed.

**DFT Calculations:** The scalar‐relativistic spin‐polarized *ab initio* calculations were performed in the framework of the full‐potential linearized augmented plane‐wave method (FP‐LAPW) as implemented in the Elk code.^28^ Structure relaxation was not performed, and relativistic effects were not taken into account. Electron correlations of the Mn 3d‐electrons were treated by assigning energy penalties *U* and *J* within the LSDA+*U* 29 formalism. The fully localized limit (FLL) double counting scheme was applied with the on‐site Coulomb repulsion term *U* = 6 eV and the Hund exchange parameter *J* = 0.9 eV. These parameters were optimized for the antiferromagnetically ordered MnO, which has an experimental magnetic moment of 4.58–4.79 µ_B_.^17^ Our test calculations have yielded 4.63 µ_B_/f. u. in MnO. In addition, we confirmed that varying the *U*_d_ =*U* – *J* value by up to 0.5 eV in Mn(OH)Cl did not make a qualitative difference to our results. Only collinear out‐of‐plane spin configurations were analyzed for four Mn atoms within a unit cell of Mn(OH)Cl. A non‐spin‐polarized self‐consistent calculation was performed first, and then used to start the spin‐polarized ones with various spin configurations. All considered scenarios of magnetic ordering have resulted in an insulating state. The total of 384 *k*‐points were used. The convergence criteria were set to 10^–6^ for the Kohn–Sham potential and field, and to 10^–4^ for the total energy. For the sake of comparison, we have conducted the same set of calculations within the generalized gradient approximation (GGA) that yielded qualitatively similar results.

**Magnetic Characterization:** Coarse‐grained powder (total mass of 27.4 mg) was placed into a gelatin capsule and fixed with cotton under argon conditions. The magnetic properties were analyzed on a CRYOGENIC Cryogen Free Measurement System (CFMS). Temperature‐dependent susceptibility measurements were conducted in a temperature range from 2 to 300 K with the cooling and heating rates of ca. 2 K/min under an external magnetic field of 0.1 T. Each measurement sequence consisted of zero‐field‐cooling (*zfc*) and field‐cooled warming (*fc*) sweeps. For the field‐dependent measurements, the sample was exposed to the magnetic flux densities between –9 T and 9 T with the sweep rates of 0.25 T/min at various temperatures. All measured data were recorded using a Vibrating Sample Magnetometer (VSM, 21 Hz).

## Supporting information

Supporting Information

## References

[1] W. Feitknecht , H. R. Oswald and H. E. Forsberg , Chimia, 1959, 13, 113.

[2] H. R. Oswald and W. Feitknecht , Helv. Chim. Acta, 1961, 44, 847–858.

[3] Y. Cudennec , A. Riou , Y. Gérault and A. Lecerf , J. Solid State Chem., 2000, 151, 308–312.

[4] H. D. Lutz , K. Beckenkamp and S. Peter , Spectrochim. Acta 1995, A51, 755–767.

[5] S. Peter and H. D. Lutz , Z. Anorg. Allg. Chem., 1998, 624, 1067–1077.

[6] Y. Iitaka , S. Locchi and H. R. Oswald , Helv. Chim. Acta, 1961, 44, 2095–2103.

[7] R. E. Dinnebier , I. Halasz , D. Freyer and J. C. Hanson , Z. Anorg. Allg. Chem., 2011, 637, 1458–1462.

[8] H. Möller , K. Beckenkamp , T. Kellersohn , H. D. Lutz and J. K. Cockcroft , Z. Kristallogr., 1994, 209, 157.

[9] R. W. G. Wyckoff , in: Crystal Structures, 2^nd^ ed., Vol. 1, Interscience Publishers, Inc. 1965.

[10] E. Ribi , Über Hydroxyverbindungen des Mangans, Dissertation, Universität Bern, 1948.

[11] J. D. Tornero and J. Fayos , Z. Kristallogr., 1990, 192, 147–148.

[12] A. N. Christensen and G. Ollivier , Solid State Commun., 1972, 10, 609–614.

[13] S. Peter , J. K. Cockcroft , T. Roisnel and H. D. Lutz , Acta Crystallogr., Sect. B, 1996, 52, 423–427.

[14] W. Kagunya , R. Baddour‐Hadjean , F. Kooli and W. Jones , Chem. Phys., 1998, 236, 225–234.

[15] M. V. Ananth , S. Pethkar and K. Dakshinamurthi , J. Power Sources, 1998, 75, 278–282.

[16] T. Kohler , T. Armbruster and E. Libowitzky , J. Solid State Chem., 1997, 133, 486–500.

[17] F. Tran , P. Blaha , K. Schwarz and P. Novák , Phys. Rev. B, 2006, 74, 155108.

[18] C. Starr , F. Bitter and A. R. Kaufmann , Phys. Rev., 1940, 58, 977–983.

[19] M. McGuire , Crystals, 2017, 7, 121.

[20] J. L. Manson , Q.‐z. Huang , C. M. Brown , J. W. Lynn , M. B. Stone , J. Singleton and F. Xiao , Inorg. Chem., 2015, 54, 11897–11905. 26645988 10.1021/acs.inorgchem.5b02162 PMC4784262

[21] J. N. McElearney , S. Merchant and R. L. Carlin , Inorg. Chem., 1973, 12, 906–908.

[22] R. D. W. Kemmitt and R. D. Peacock , in: The Chemistry of Manganese, Technetium and Rhenium,, Pergamon Press, Oxford, UK, 1973.

[23] H. Huppertz , Z. Kristallogr., 2004, 219, 330–338.

[24] Topas, 4.2, Bruker Analytical X‐ray Instruments Inc., Madison, Wisconsin, USA, 2009.

[25] Sadabs‐2014/5, Bruker AXS, Madison, Wisconsin (USA), 2015.

[26] L. Krause , R. Herbst‐Irmer , G. M. Sheldrick and D. Stalke , J. Appl. Crystallogr., 2015, 48, 3–10. 26089746 10.1107/S1600576714022985 PMC4453166

[27] Opus, v.7.2, Bruker Optik GmbH, Billerica, MA, USA, 2012.

[28] The Elk Fp‐lapw Code, *version 3.1.12*, **2010**, http://elk.sourceforge.net.

[29] J. P. Perdew and Y. Wang , Phys. Rev. B, 1992, 45, 13244–13249. 10.1103/physrevb.45.13244 10001404

