# Supplementary material for: Synthesis and Characterization of the New Manganese Hydroxide Chloride γ‐Mn(OH)Cl
Source: Eur J Inorg Chem. 2018 Nov 6;2018(42):4630–7. doi: 10.1002/ejic.201800928 (PMC6334172; doi:10.1002/ejic.201800928)
Supplement: Supplementary file 1 — Supporting Information [file EJIC-2018-4630-s001.pdf]

European Journal of Inorganic Chemistry

Supporting Information

# Synthesis and Characterization of the New Manganese Hydroxide Chloride γ-Mn(OH)Cl

Viktoria Falkowski, Alexander Zeugner, Anna Isaeva, Michael Ruck, and Hubert Huppertz*

ejic201800928-sup-0001-SupMat.pdf

Table of Contents

1. Bond angles derived from single-crystal data
2. X-ray powder diffraction

# 1. Bond angles derived from single-crystal data

**Table S1:** Bond angles (/°) in *γ*-Mn(OH)Cl (standard deviations in parentheses).

| | | | |
|---|---|---|---|
| $O–Mn–O^{i}$ | 110.72(1) | $O–Mn–Cl^{iii}$ | 165.15(3) |
| $O–Mn–O^{ii}$ | 82.23(3) | $O^{i}–Mn–Cl^{iv}$ | 165.15(3) |
| $O^{i}–Mn–O^{ii}$ | 82.23(3) | $O^{ii}–Mn–Cl$ | 179.10(3) |
| $Cl–Mn–Cl^{iii}$ | 87.11(2) | $Mn^{vi}–O–Mn$ | 110.72(5) |
| $Cl–Mn–Cl^{iv}$ | 87.11(2) | $Mn^{vi}–O–Mn^{ii}$ | 97.77(3) |
| $Cl^{iii}–Mn–Cl^{iv}$ | 83.45(2) | $Mn–O–Mn^{ii}$ | 97.77(3) |
| $O^{i}–Mn–Cl^{iii}$ | 82.62(2) | Mn–O–H | 116.2(6) |
| O–Mn–Cl | 97.27(3) | $Mn–O^{i}–H^{i}$ | 116.2(6) |
| $O^{i}–Mn–Cl$ | 97.27(3) | $Mn–O^{ii}–H^{ii}$ | 115(2) |
| $O^{ii}–Mn–Cl^{iii}$ | 93.56(2) | | |

(i) *x*, 1+*y*, *z*; (ii) 2-*x*, 0.5+*y*, 1-*z*; (iii) 1-*x*, 0.5+*y*, 1-*z*; (iv) 1-*x*, -0.5+*y*, 1-*z*; (v) 2-*x*, -0.5+*y*, 1-*z*; (vi) *x*, -1+*y*, *z*.

## 2. X-ray powder diffraction

Figure S1 shows the comparison of the experimental powder pattern to the theoretical pattern derived from single-crystal data. The title compound $\gamma$-Mn(OH)Cl represents the main phase with an unidentified side phase and molybdenum as remains of the capsule. The lattice parameters derived from powder diffraction data deviate only slightly from the obtained values from the single-crystal structure determination (see Tab. 1).

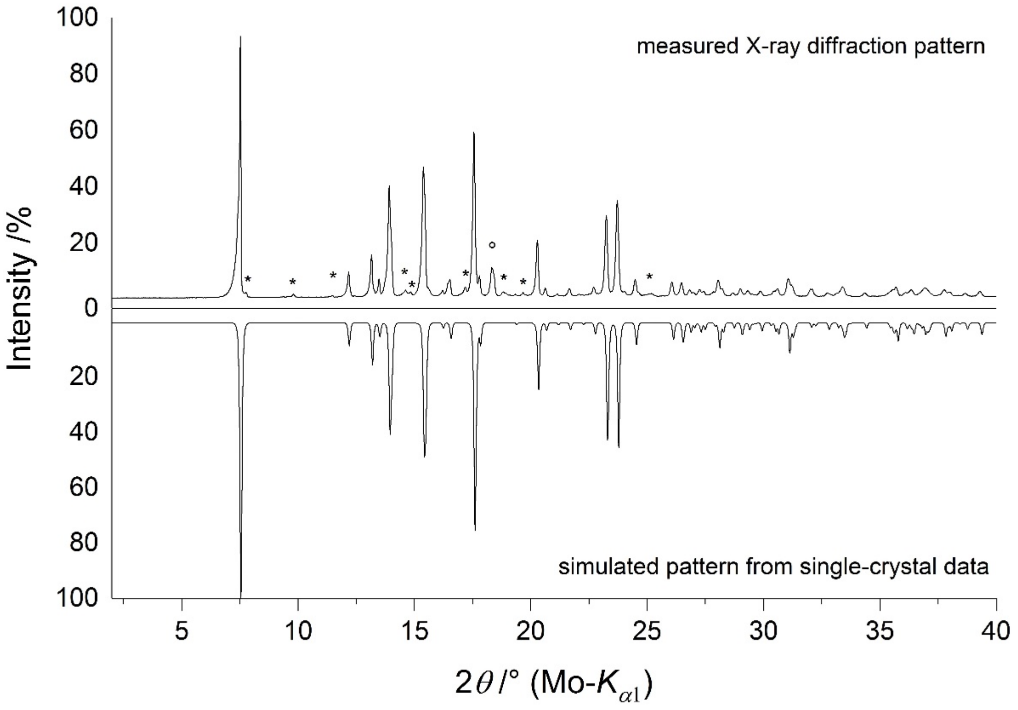


**Figure S1:** Experimental powder pattern (top) compared to the simulated powder pattern of $\gamma$-Mn(OH)Cl (bottom) calculated from the single-crystal data; reflections marked with an asterisk originate from an unknown side phase, the reflection marked with a circle represents remains of the molybdenum capsule.
